# Towards the Understanding of the Function of Lanthipeptide and TOMM-Related Genes in *Haloferax mediterranei*

**DOI:** 10.3390/biology12020236

**Published:** 2023-02-02

**Authors:** Thales Costa, Elena Cassin, Catarina Moreirinha, Sónia Mendo, Tânia Sousa Caetano

**Affiliations:** 1 CESAM and Department of Biology, University of Aveiro, 3810-193 Aveiro, Portugal; 2 CESAM and Department of Chemistry, University of Aveiro, 3810-193 Aveiro, Portugal

**Keywords:** gene deletion, lantibiotic, class halobacteria, halophilic archaea, antimicrobials, gene clusters, biosynthesis

## Abstract

**Simple Summary:**

The knowledge of the biosynthetic pathways and the biological role of secondary metabolites produced by the third domain of life is limited when compared with bacteria or eukaryotes. Herein, we investigated, in more detail, genes encoding enzymes that are predicted to be involved in the biosynthesis of peptide secondary metabolites in halophilic archaea regarding (i) their transcription, (ii) their association with antimicrobial activity, and (iii) the impact of their absence on the biomolecular profile of the cells.

**Abstract:**

Research on secondary metabolites produced by Archaea such as ribosomally synthesized and post-translationally modified peptides (RiPPs) is limited. The genome of *Haloferax mediterranei* ATCC 33500 encodes lanthipeptide synthetases (*med*M1, *med*M2, and *med*M3) and a thiazole-forming cyclodehydratase (*ycaO*), possibly involved in the biosynthesis of lanthipeptides and the TOMMs haloazolisins, respectively. Lanthipeptides and TOMMs often have antimicrobial activity, and *H. mediterranei* has antagonistic activity towards haloarchaea shown to be independent of *med*M genes. This study investigated (i) the transcription of *ycaO* and *med*M genes, (ii) the involvement of YcaO in bioactivity, and (iii) the impact of YcaO and MedM-encoding genes’ absence in the biomolecular profile of *H. mediterranei*. The assays were performed with biomass grown in agar and included RT-qPCR, the generation of knockout mutants, bioassays, and FTIR analysis. Results suggest that *ycaO* and *med*M genes are transcriptionally active, with the highest number of transcripts observed for *med*M2. The deletion of *ycaO* gene had no effect on *H. mediterranei* antihaloarchaea activity. FTIR analysis of *med*M and *ycaO* knockout mutants suggest that MedMs and YcaO activity might be directly or indirectly related t lipids, a novel perspective that deserves further investigation.

## 1. Introduction

In recent years, ribosomally synthesized and post-translationally modified peptides (RiPPs) have been emerging as an interesting source of novel natural products, with diverse chemical structures and functions [1]. RiPPs are divided into classes according to the nature of the post-translational modification enzymes (PTMs) that produce a modified and functional peptide [2]. Briefly, and considering the general biosynthetic mechanism, these enzymes recognize a cognate precursor peptide by binding to the leader peptide and modifying the amino acids of the core peptide in subsequent reactions [1]. At the genetic level, the biosynthetic gene clusters (BGCs) are identified by many bioinformatic approaches, which search for the presence of a gene(s) encoding the core PTM enzyme(s) characteristic of each subtype of RiPP [3]. Within the domain Archaea, BGCs encoding the biosynthesis of two subtypes of RiPPs, the lanthipeptides and TOMMs (thiazole/oxazole-modified microcins), were identified in haloarchaea, including in the *Haloferax mediterranei* ATCC 33500 strain [4,5]. 

**Figure 1:**
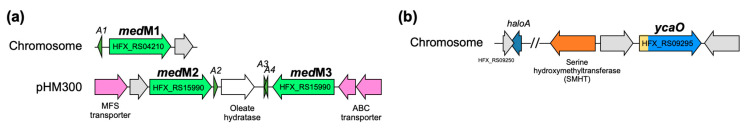
BGCs of lanthipeptides (**a**) and haloazolisin (**b**) found in *H. mediterranei* ATCC 33500 genome. Grey ORFs have unknown functions. A1-A3 represent putative *medA* structural genes and *halo*A encodes the structural gene predicted by [4] as haloazolisin PP. The N-terminal of *ycaO* gene (yellow) encodes a barely recognizable C-protein domain.

As suggested by the name, lanthipeptides are RiPPs that contain (methyl)lanthionine residues, and the enzymes responsible for their installation define their classification. The BGCs of *H. mediterranei* encode three class II synthetases, generally named LanMs (Figure 1a). LanMs are bifunctional enzymes since they catalyze the dehydration of Ser and Thr to Dha and Dhb, respectively, and the cyclization reaction between Cys and Dha or Dhb to form lanthionine (Lan) or methyllanthionine (MeLan) residues, respectively [6]. In *H. mediterranei*, the three *lan*M genes were found in the chromosome (*medM1*) and the pHM300 megaplasmid (*medM2* and *medM3*; Figure 1a). The possible relation of lanthipeptide production and the *H. mediterranei* antihaloarchaea activity was investigated by testing *med*M knockout mutants and it was concluded that they are not drivers of such inhibitory ability [5]. However, at the time, it was not possible to confirm that they were not haloarcheocins since it is unknown if *med*M BGCs are cryptic. 

TOMMs are a subtype of RiPPs containing thiazole or (methyl)oxazole heterocycles and the core biosynthetic enzyme of TOMMs is a cyclodehydratase from the YcaO superfamily, also known as the D-protein [7]. The YcaO cyclodehydratases catalyze the cyclodehydration of Cys in thiazolines, and Ser or Thr in oxazolines or methyloxazolines, respectively [8]. YcaO cyclodehydratases normally work together with a C-protein, which enhances their activity. They can be encoded in the same gene, producing a bifunctional enzyme, or in separate genes [7]. *H. mediterranei* encodes a TOMM cluster of the haloazolisin’s family in its chromosome, which contains a fused cyclodehydratase (herein *ycaO*) with a barely recognizable C-protein domain [4] (Figure 1b). 

The main objective of this work was to add information to the current, but scarce, knowledge on *H. mediterranei* RiPPs, as a case study of the haloarchaea group. More specifically, we intended to (i) determine if the *med*M and *ycaO* genes were cryptic in the conditions where the antihaloarchaea activity is detected (agar media growth), (ii) test the TOMM BGC relation with the antagonistic effect, and (iii) understand the effect, at the biomolecular level, of the absence of the YcaO and MedMs-encoding genes. 

## 2. Materials and Methods

### 2.1. Culture Media

The strain *H. mediterranei* WR510 (*H. mediterranei* Δ*pyrE*) and its derivatives were routinely grown in YPC agar or YPC broth (Table S1) at 37 °C for the time required for each assay. *Halobacterium salinarum* NRC-1 was cultivated in YPCss agar or YPCss broth (Table S1) at 45 °C for 5 days or 37 °C for 7 days. 

### 2.2. Generation of Knockout Mutants

The knockout of the *ycaO* gene was generated using the *H. mediterranei* WR510 strain as described by [9] and [5] using knockout plasmids based on the plasmid pTA131 [10]. As such, a knockout plasmid, pKO_ycaO, was constructed to allow the deletion of *ycaO* from the *H. mediterranei* WR510 genome by recombination. The plasmids were constructed via the insertion of the two flanking regions of *ycaO* into the *lacZ* of pTA131. The amplification of each flanking region was performed in a final volume of 50 μL with the 2X Platinum SuperFi PCR Master Mix (Thermo Scientific), following the manufacturer’s instructions and using the primers and annealing temperature listed in Table S2. The amplicons were purified with the NZYGelpure Kit (NZYTech, Portugal) and digested with the corresponding Anza restriction enzymes (Thermo Scientific; Table S2) in a final volume of 20 µL, according to the manufacturer’s instructions. The pTA131 was also digested in the same conditions. After digestion, the DNA was analyzed and recovered from the agarose gel with the NZYGelpure Kit (NZYTech, Portugal). Ligation was performed in a 20 µL reaction containing 5 μL of Anza T4 DNA Ligase Master Mix (Thermo Scientific), 50 ng of digested vector, 40 ng of the digested up fragment, and 40 ng of the digested down fragment. The ligation was incubated at 37 °C for 20 min and 5 μL were transformed by heat shock into 50 μL of *E. coli* NZY5α competent cells (NZYTech, Portugal). The transformants were selected on LB agar plates containing 50 μg/mL of ampicillin and 0.2 mg/mL of X-Gal, overnight at 37 °C. The screening of white colonies was performed by colony PCR using universal primers (*lac*Z MCS; Table S2). The expected amplicon size for colonies containing the recombinant plasmid was approximately 1900 bp, corresponding to the *lacZ* gene with the insertion of both up and down fragments. A positive colony was selected to extract pKO_ycaO with the NZYMiniprep kit (NZYTech, Portugal), and its MCS region was sequenced at StabVida (Portugal).

*H. mediterranei* was transformed with pKO_ycaO using the transformation PEG-mediated protocol described by [10] (Figure S2) and the pop-in transformants were selected in CAM-agar plates (Table S1), at 37 °C for 4 days. Confirmation of pop-in transformants and generation of pop-out transformants was performed as described [5,9]. Pop-out transformants were screened by PCR with the *ycaO* flanking region ycaO_UP_FW and ycaO_DOWN_RV primers (Table S2) to select pop-out knockout mutants, which were further confirmed by absolute qPCR targeting the *ycaO* deleted gene. The reactions were performed with the 2X PowerUp SYBR Green Master Mix (Thermo Fisher) as described by [5], with the primers listed in Table S2. 

### 2.3. Viable Cells Count and Anti-Haloarchaea Activity

Liquid cultures of *H. mediterranei* WR510 or the knockout mutants were prepared in 5 mL of YPC broth and grown at 37 °C for 48 h, with aeration (180 rpm). Their optical density at 600 nm (OD_600_) was adjusted to 0.02, and 25 μL of this culture was plated as a “dot” in the center of YPC-agar plates (Figure S3) that were incubated at 37 °C for 5 days. 

For viable cell counts, one plate of each biological replicate of *H. mediterranei* WR510 was collected every day, and the number of viable cells in the dot biomass was quantified by resuspension of the dot in four mL of YPC broth. The OD_600_ was measured, and viable cells were determined by counting CFU/mL as described by [11] and using four biological replicates. For the knockout of *H. mediterranei ∆ycaO*, the protocol applied was the same, but viable counts were performed after 4 days post-plating.

Two plates of each biological replicate of the *H. mediterranei* WR510 were collected for the bioactivity assay, and plates were treated for 15 min with UV light and conserved at 4 °C. On day 5, all the plates were used for overlay bioassay with the indicator strains *H. salinarum* or *H. volcanii*. The two indicator strains were grown in the appropriate YPC broth (Table S1) for 7 and 5 days, respectively, at 37 °C. The OD_600_ of the cultures was measured and added to 15 mL of the respective soft agar (Table S1), at a final OD_600_ = 0.03. Each indicator culture was then poured onto the *H. mediterranei* plate and incubated at 37 °C until the halo of inhibition was clearly visible for measuring. The same protocol was followed to test the bioactivity of the knockout *H. mediterranei ∆ycaO*, and the protocol applied was the same. However, viable counts were performed only after 4 days post-plating.

### 2.4. Preparation of plates for RNA extraction and FTIR analysis

The *H. mediterranei* strains were grown in 5 mL of YPC broth and incubated at 37 °C for 72 h, with aeration (180 rpm). The OD_600nm_ was measured, and the cultures were diluted in YPC for a final OD_600nm_ equal to 0.02 and a final volume of 1 mL. Plates were prepared in 15 mm Petri dishes containing approximately 10 mL of YPC agar and 25 µL of the cultures prepared, and spotted in the center of the plate (Figure S4). The plates were incubated at 37 °C for the time determined by the subsequent analysis (RNA extraction or FTIR). Five biological replicates were prepared for each strain.

### 2.5. RNA Extraction and cDNA Synthesis

Total RNA was extracted after 24, 48, and 72 h of growth of the strain *H. mediterranei* WR519 as above described. The RNA was extracted with the GRiSP Total RNA Kit—Bacteria, according to the manufacturer’s instructions and starting with resuspension of the biomass in the plates in 1 mL of YPC broth (24 h of growth). For the cultures grown for 48 and 72 h, the biomass was resuspended in 2 mL of YPC broth and the OD_600nm_ was measured. The suspensions were further diluted in YPC broth to achieve an OD_600nm_ of 0.5, and 1 mL was used for the RNA extraction so as not to exceed the maximum number of cells established by the manufacturer. RNA integrity was subsequently evaluated through agarose gel electrophoresis and RNA concentration was determined with Qubit (Invitrogen). 

The total RNA was used to synthesize cDNA with the SuperScript VILO cDNA synthesis kit (Thermo Fisher Scientific). Prior to the synthesis, the total RNA samples were treated with DNase to remove any DNA contamination as follows: 1 μL of 10X ezDNase buffer, 1 μL of ezDNase, and varied volumes of RNA to allow the use of 250 ng and nuclease-free water up to a final volume of 10 μL. The reactions were mixed and incubated at 37 °C for 2 min. Then, the reactions were centrifuged and placed on ice, and for each reaction, a volume of 4 μL of SuperScript IV VILO Master Mix and 6 μL of nuclease-free water was added. The reverse transcription reaction was performed following the manufacturer’s instructions, and the cDNA was stored at −80 °C.

### 2.6. Transcriptional Analysis by qPCR

The synthesized cDNAs were used to quantify the gene transcripts of genes of the lanthipeptides and haloazolisin BGCs (*med*M1, *med*M2, *med*M3, and *ycaO*) by absolute qPCR quantification. The *rpl16* transcripts, which encode the 50S ribosomal protein L16, were also quantified to use as a reference gene in normalization, as described by [12]. This strategy requires a quantification curve, which was obtained with a 10-fold dilution series of total DNA from *H. mediterranei* WR510, ranging from 1.73 × 10^5^ to 1.73 × 10^1^ copy number/μL (considering the target gene) as a DNA template. Each qPCR reaction had a final volume of 10 μL consisting of 5 μL of PowerUp^TM^ SYBR^TM^ Green Master Mix (Thermo Fisher Scientific), 0,5 μL of the forward primer (10 pmol/μL), 0,5 μL of the reverse primer (10 pmol/μL; Table S2), 1 μL of the template (either the diluted total DNA or a 1/10 dilution of the cDNA to test), and 3 μL of DNase-free water. Amplification was performed with the CFX96 real-time PCR system (Bio-Rad, Hercules, CA) as follows: 50 °C for 2 min, 95 °C for 2 min, and 40 cycles consisting of 95 °C for 15 s and 57–61 °C for 1 min (Table S2), followed by plate reading. Upon completion, a melting curve was obtained by submitting the amplicons to a temperature range from 65 °C to 95 °C, with an increase of 0.5 °C at 5 s intervals, in order to evaluate non-specific amplification. The reaction efficiency, R^2^, and quantification were calculated by the CFX Manager software (Bio-Rad), and only reactions with efficiencies of 90–110% were considered for analysis. Five biological and two technical replicates were performed for each condition tested.

### 2.7. FTIR Analysis

Strains of *H. mediterranei* WR510, Δ*M1M2M3* [5], and Δ*ycaO* (this study) were grown in plates as mentioned above. The colonies were analyzed by FTIR on the 1st, 6th, and 14th days post-plating. The analyses were conducted in the ALPHA Platinum ATR-FTIR (Bruker, Germany) at wavenumbers ranging from 4000 cm^−1^ to 500 cm^−1^ and controlled temperature (23 °C) and humidity (35%). The procedure involved the collection of the colonies with a loop to place them at the center of a crystal with a 2 mm × 2 mm horizontal single-reflection diamond, followed by drying with a cold air flow to reduce the influence of water in the spectra. The reflection diamond was cleaned with ethanol 70% and distilled water between readings. In order to better visualize the results, multivariate analysis was used. The spectra, which were obtained in the OPUS format, were converted to JCAMP.DX and analyzed by principal component analysis (PCA) using an in-house developed data analysis package—CATS build 97. Principal component analysis (PCA) was used to find the major sources of variability in the data and detect outliers and the probable presence of clusters. Before PCA, the spectra were standard normal deviation (SNV)-corrected. The data were then used to construct graphics representing the score scatter and the resultant loadings profile with the GraphPad Prism 8.0.2.

### 2.8. Bioinformatic Analysis

The classification of protein families and the prediction of their putative roles were investigated with the InterPro (EMBL-EBI). The genetic regions of the *ycaO* gene (minus 10 kb and plus 10 kb) in different halobacteria were retrieved from the NCBI database, after tblastn search of YcaO from *H. mediterranei* in Microbial Blast (NCBI). The alignment of the genetic regions was performed with clinker [13], considering an identity threshold between groups of genes of 0.3. The number of genomes available for haloarchaea was based on the information available in the NCBI Microbial Genomes database (accessed on 9 November 2022). The sequence similarity network was performed with the EFI-EST tool [14] using the families option (IPR003776) and an E-value of 1. The final network was visualized with Cytoscape 3.9.1. [15].

## 3. Results and Discussion

### 3.1. Characterization of H. Mediterranei Growth and Antihaloarchaeal Activity on YPC Agar

**Figure 2:**
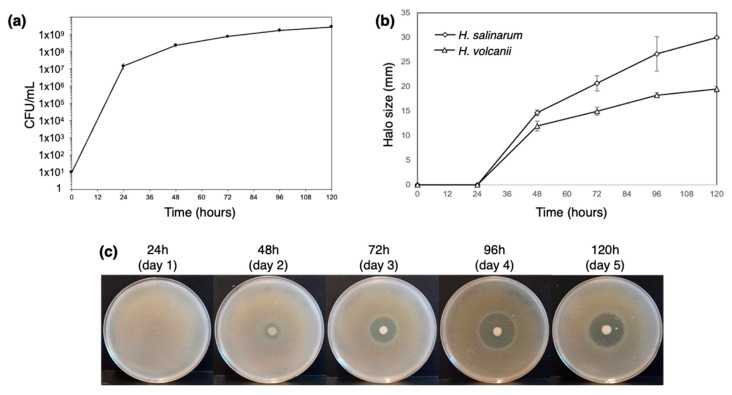
Characterization of *H. mediterranei* growth and antihaloarchaea activity in YPC agar for 5 days. The number of viable cells in the dot biomass growing in agar plates (**a**) and the size of their inhibition halos against the haloarchaea *H. salinarum* and *H. volcanii* (**b**). The increase in the halos against *H. salinarum* is also shown, as a visual example of inhibition (**c**).

The antimicrobial activity of *H. mediterranei* ATCC 33500 has been characterized primarily in agar media, and it is very reduced, or even absent, in broth. However, the production of biomass has been evaluated exclusively in broth. Therefore, we considered it important to establish protocols that evaluate, for instance, growth and gene transcription, just to name a few, with cultures in solid media. Thus, we started by characterizing *H. mediterranei* growth and antimicrobial activity on YPC agar over 5 days. The protocol of [11] was applied for the first time to *H. mediterranei* WR510, and the dilution of 1 × 10^−4^ was identified as the most appropriate for CFU counting. The growth curve obtained (Figure 2a) showed that the number of viable cells increased exponentially within the first 24 h (1.48 × 10^7^ ± 5.01 × 10^6^ CFUs/mL) and continued to slowly increase up to 48 h (8.13 × 10^7^ ± 5.44 × 10^6^ CFUs/mL). In the following three days, the number of viable cells stabilized until reaching 2.31 × 10^8^ ± 5.31 × 10^7^ CFUs/mL. This was similar to that obtained when *H. mediterranei* WR510 was cultivated in YPC broth since it entered the stationary phase after 32 h of growth [5]. The inhibition halos were measured to understand the increase in the antimicrobial activity of *H. mediterranei* during the 5 days (Figure 2b,c). In the first 24 h, no inhibition was evident against any of the indicator strains (Figure 2b). Then, the inhibitory activity strongly increased between 24 and 96 h (Figure 2b) and eventually stabilized between 96 h and 120 h (Figure 2b). Thus, the largest halos against both indicators were observed after 4 or 5 days of incubation. Even so, this type of assay does not allow us to clarify whether the increase in inhibition corresponds to a higher concentration of the antimicrobial compound(s) or whether it results from a greater diffusion of the antimicrobial compound(s) over time.

### 3.2. Transcriptional Analysis of the Biosynthetic Enzymes of RiPPs

**Figure 3:**
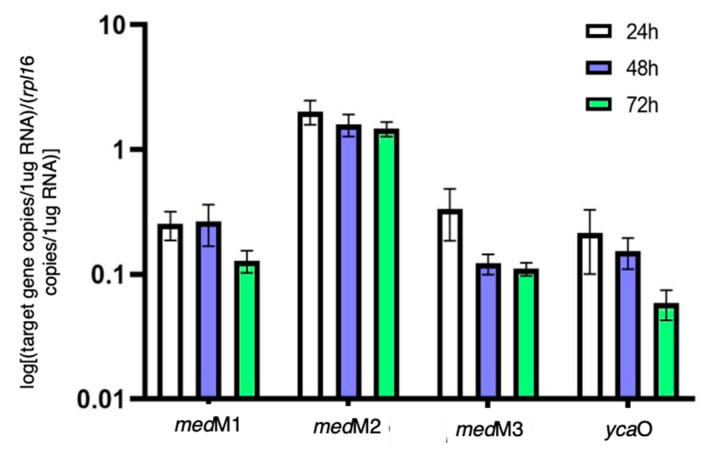
Quantification of transcripts encoding the lanthipeptide synthetases and the YcaO protein in *H. mediterranei* YPC agar cultures over three days, normalized with the gene *rpl*16 copy number.

As aforementioned, previous studies suggested that the two lanthipeptide clusters of *H. mediterranei* were not associated with the production of lantibiotics [5]. This is because the deletion of *med*M1, *med*M2, and *med*M3 genes, which encode the lanthipeptide synthetases, did not affect the activity against haloarchaea. However, this does not prove that they are not involved in lanthipeptides with bioactivity, as they may, for example, be cryptic genes, as seen for many microbial secondary metabolites [16,17]. Therefore, in this study, we filled that gap by investigating the transcription of *med*M1, *med*M2., and *med*M3 genes over time. Additionally, we also quantified the transcription of the *ycaO* gene, before proceeding to the generation of its knockout mutant. The results (Figure 3) show that, on average, the genes *medM1*, *medM3,* and *ycaO* were 10X less transcribed than the *rpl16* gene, which is considered a highly expressed gene. Surprisingly, the transcripts encoding the *med*M2 were, on average, 1.8X higher than those of the *rpl16* gene. The genes *rpl16*, *haloM1,* and *ycaO* reside in the main chromosome, whereas *med*M2 and *med*M3 co-localize in the megaplasmid pHM300. Thus, the difference observed between *med*M2 and *med*M3 transcription was unexpected. Considering the time of growth, results show that the copy number of all the transcripts tested did not increase after 24 h of growth. In fact, we observed a tendency to maintain or decrease over the three days tested (Figure 3). These results indicate that all four genes are transcriptionally active under the conditions tested. As such, we proceeded with the deletion of the *ycaO* gene and the evaluation of its antihaloarchaeal activity.

### 3.3. Generation of Haloazolisin Knockout Mutants and Their Antihaloarchaeal Activity

**Figure 4:**
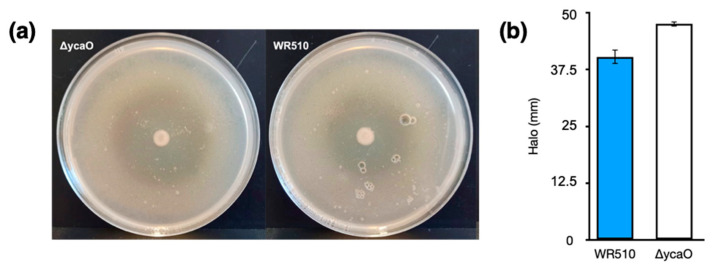
Inhibition halos of *H. mediterranei* WR510 and Δ*ycaO* against *H. volcanii* after 4 days of growth (**a**) and corresponding sizes in mm (**b**).

As abovementioned, previous studies showed that the deletion of *med*M1, *med*M2, and *med*M3 genes encoding the lanthipeptide synthetases in *H. mediterranei* did not affect its activity against other haloarchaea [5]. However, *H. mediterranei* has another putative RiPP cluster in its genome, encoding the biosynthesis of a type of TOMMs named haloazolisin [4]. As TOMMs usually have antimicrobial activity, we deleted the core biosynthetic gene *ycaO* to evaluate its involvement in the antihaloarchaea activity of *H. mediterranei*. The generation and confirmation of the Δ*ycaO* mutant followed the same stages described in [5], which involved qPCR, given its sensibility. The number of viable cells and the inhibition ability of the mutant werer assessed after four days of growth. The results showed no difference between the amount of *H. mediterranei ΔycaO* (8.15 × 10^7^ ± 1.89 ×10^7^ CFUs/mL) and *H. mediterranei* WR510 (7.42 × 10^7^ ± 2.77 × 10^6^) viable cells. Therefore, any possible difference in the antihaloarchaea activity between the two strains could not be attributed to differences in biomass. Bioassays revealed that *H. mediterranei ΔycaO* retained inhibitory activity against haloarchaea (Figure 4a), although the size of the halos was slightly larger than those recorded in the wildtype (Figure 4b). These types of differences were not observed for *H. mediterranei* lacking the three *med*M genes [5]. Though diffusion assays are not very accurate, the halos of Δ*ycaO* raised some doubts about the true role of YcaO in haloarchaea. Recently, the antagonistic activity of *H. mediterranei* was found to be promoted primarily by halolysins, which is a subtilin-like serine protease of haloarchaea [18].

### 3.4. In Silico Analysis of H. Mediterranei Haloazolisin Genetic Environment

**Figure 5:**
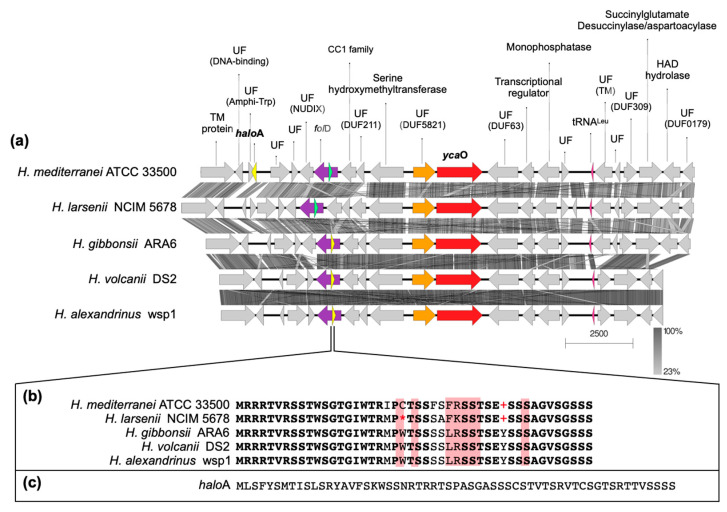
Alignment of the genetic environment of *H. mediterranei* containing the *ycaO* and the *halo*A genes with other *Haloferax* spp. (**a**), showing the function of the proteins encoded (UF stands for unknown function and, if identified, the reference to the domain is also given). The genes encoding haloazolisin’s PPs predicted by [4] are highlighted in yellow (**a**) and the amino acid sequences are shown in (**b**,**c**). A gene encoding a PP similar to those found in *H. gibbonsii*, *H. volcanii,* and *H. alexandrinus* was also identified in *H. mediterranei* and *H. larsenii* (green ORF in (**a**) and respective amino acid sequence in (**b**)), but its sequence has UAG (+) and UGA (*) codons, which are termination codons.

**Figure 6:**
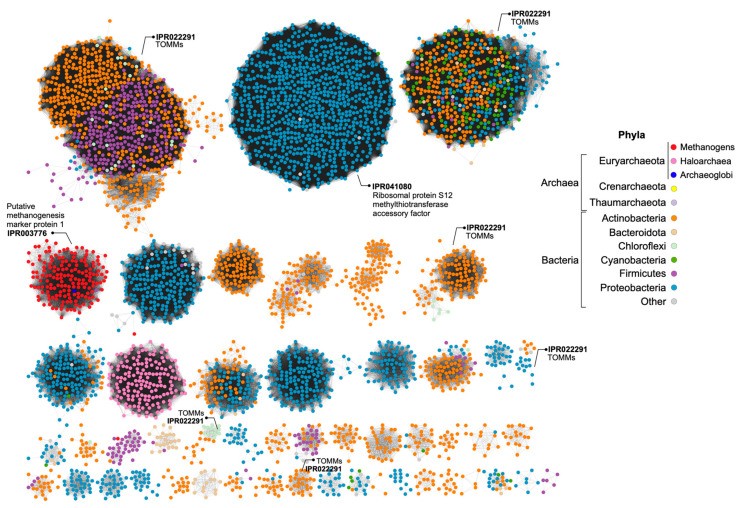
Sequence Similarity Network (SSN) of proteins containing the YcaO-like domain (IPR003776), where each node represents a protein encoded in the genome of Archaea or Bacteria. Groups of proteins with other domains identified as being involved in the production of RiPPs (TOMMs) are marked, as well as groups with YcaO proteins not involved in RiPPs biosynthesis (methanogens—IPR003776 and IPR041080). YcaOs of haloarchaea is found in a separate group of uncharacterized proteins. Singletons and groups with a low number of nodes were excluded.

The BGCs encoding the biosynthesis of lanthipeptides of *H. mediterranei* were previously analyzed in detail by [5]. At the time of their description, haloazolisins were defined as TOMM BGCs from haloarchaea harboring a fused cyclodehydratase with a small C-protein domain (herein YcaO) and a recognizable precursor peptide (herein *halo*A) [4]. In *H. mediterranei*, the proposed *halo*A gene was found eight genes upstream of the *ycaO* gene (Figure 5a,c). The genes immediately upstream and downstream of the *ycaO* gene encode proteins of unknown function (Figure 5a). The upstream and downstream regions of the *ycaO* gene of different haloarchaea were aligned in order to detect genes that can possibly compose the haloazolisin BGC (Figure S5). Results show that the genetic environment of *ycaO* varies, but with a tendency to be very similar within species of the same genus (e.g., *Haloferax*), especially the upstream region that includes an ORF encoding a protein of unknown function (immediately before *ycaO*) and an ORF encoding a serine hydroxymethyltransferase (SHMT) (Figure 5a). In fact, these two genes are found in all haloarchaeal genomes, even if not genetically close to *ycaO*. The array downstream of *ycaO* is more variable, but tblastn searches against available haloarchaeal genomes showed that they are conserved in haloarchaea, but not always located in proximity to *ycaO*. Focusing on the strains from the *Haloferax* genus, the *H. mediterranei* PP predicted by [4] is dissimilar to the ones predicted for *H. gibbonsii*, *H. volcanii,* and *H. alexandrinus*, which are encoded and overlapped with the *fol*D gene (Figure 5). However, a homologous ORF was found in the *fol*D region of *H. mediterranei* and *H. larsenii* (green ORFs in Figure 5). These ORFs were likely not previously considered since they possess the UAG and UGA codons, which are translational termination codons (Figure 5b). However, according to the codon usage table, the UAG is the weakest STOP codon of *H. mediterranei*. Therefore, comparative analysis of the *ycaO* genetic region among haloarchaea did not allow the identification of putative haloazolisin BGC constituents as has been possible for other RiPPs [5,19,20]. Moreover, the *ycaO* gene, as well as its surroundings, is found in all haloarchaeal genomes, a characteristic that is not seen in lanthipeptides BGCs [5] or the majority of RiPPs BCGs. In fact, the YcaO protein could be involved in the biosynthetic pathway found in all haloarchaea since it is encoded in all haloarchaeal genomes (354 proteins in 316 genomes according to InterPro). The YcaO superfamily is vast, and the functions of most of these proteins are still unknown. However, they are not always associated with the biosynthesis of RiPPs [21]. For instance, Ec-YcaO seems to be a scaffold protein that enhances the RimO-dependent β-thiomethylation of *E. coli* ribosomal protein S12 [22] (Figure 6). In Archaea, a YcaO is involved in the thioamidation of the active site of the methyl-coenzyme M reductase, which is essential for methanogenic and methanotrophic strains [23]. However, members of this family (IPR017667) represent a distinct clade among proteins with the YcaO-like domain (IPR003776), which does not include the YcaOs found encoded in haloarchaeal genomes (Figure 6). 

### 3.5. FTIR Analysis of H. Mediterranei Lacking RiPPs Core Biosynthetic Enzymes

**Figure 7:**
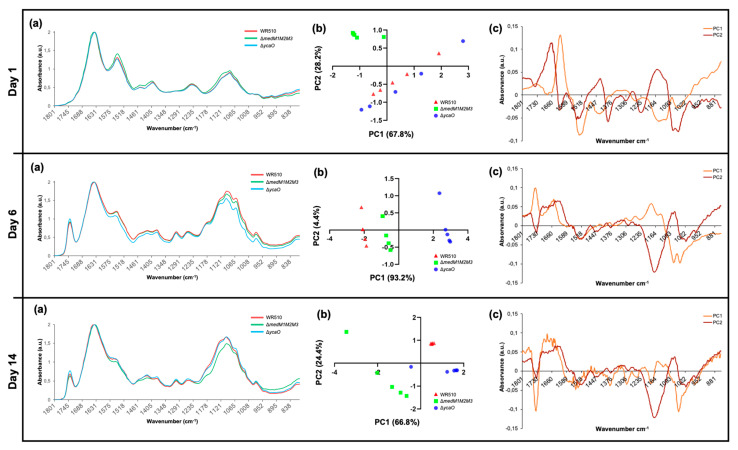
Results of FTIR analysis of *H. mediterranei* WR510, Δ*medM1M2M3,* and Δ*ycaO* strains grown in YPC agar plates over 14 days. Panel (**a**) shows the general spectra obtained (mean of all replicates), while panels (**b**,**c**) present the results of PCA analysis. (**b**) Scores scatter plot; (**c**) loadings plot profile.

From what we know, LanM proteins have been always involved in pathways generating active RiPPs. However, as discussed previously, YcaO proteins can have other biological roles in addition to the biosynthesis of RiPPs. FTIR identifies chemical bonds in a molecule by producing an infrared absorption spectrum and can therefore generate a biochemical profile of microorganisms, considered to be a highly specific fingerprint that enables accurate microbial identification and the analysis of cell components [24,25,26,27]. It is an easy-to-perform technique using bacterial colonies, while being cheap (no reagents needed) and delivering fast results. Since it was proven to be highly sensitive and reproducible, it has been widely used for the identification and differentiation of bacteria, especially clinically relevant strains and foodborne pathogens [28,29,30]. Studies applying FTIR to halophilic archaea are scarce but have proven to be useful for specimen differentiation [31]. Therefore, in order to understand whether the absence of MedMs and YcaO impacted the biomolecular profile *H. mediterranei*, the Δ*medM1M2M3* and Δ*ycaO* knockout mutants were analyzed by FTIR over 14 days (Figure 7a), and the spectra obtained were interpreted based on PCA analysis (Figure 7b,c) performed for the infrared region between 1800 and 800 cm^−1^, which was the more suitable for our study as it included protein, lipids, and fingerprint vibrations. The PCA analysis of the first day of growth showed that Δ*medM1M2M3* is the most distinct group, located in the upper left quadrant, while Δ*ycaO* and WR510 were scattered along the remaining quadrants (Figure 7b). The wavenumbers, which better explain the Δ*medM1M2M3* distribution, were identified in the loadings plot profile (Figure 7c) to be 1150 cm^−1^, 1450/1550/1650 cm^−1^, and 1280 cm^−1^, which can be assigned to the vibration of phosphodiester bonds, amides (III, II, and I) from proteins, and lipids (including fatty acids) (Figure 7c, Table 1). On the sixth day, Δ*ycaO* was the most distinct group, located primarily in the bottom right quadrant, while Δ*medM1M2M3* and WR510 occupied the upper and bottom left quadrants. Such separation is primarily characterized by a peak around 1750 cm^−1^ and another at 1650 cm^−1^ (Figure 7b) and wavenumbers ranging from 1137–1145 cm^−1^ and 1180–1190 cm^−1^ (Figure 7c, Table 1). These represent C=O bonds and C=C bonds, respectively, from lipids (including fatty acids), phosphate, and/or oligosaccharides, amides, and/or deoxyribose (Table 1).

Intriguingly, the strain Δ*ycaO* was shown to be the most distinctive strain at this time point of the analysis. This shift can possibly be explained by the fact that, over time, discrepancies between Δ*ycaO* and other groups could be more significant than the differences between Δ*medM1M2M3* and other strains. PCA analysis of the results after two weeks of growth showed that most of the samples of the same strain clustered within a unique quadrant, indicating that all of them differ from each other at the molecular level (Figure 7b). At this time point, Δ*ycaO* was located primarily at the bottom right quadrant, Δ*medM1M2M3* occupied primarily the bottom left quadrant, and all samples from the WR510 group were located in the upper right quadrant with all the replicates forming a very isolated, small cluster (Figure 7b). The control strain, WR510, is marked by a predictable distinction at the phosphodiester bond level (Figure 7c). Furthermore, both Δ*medM1M2M3* and Δ*ycaO* differ from WR510, as they are located in the bottom left quadrant, distinguished by a large peak at approximately 1740 cm^−1^ characterized by contributions of C=O and/or C=C from lipids. When compared to day 6, Δ*ycaO* lost its notable variation in the wavenumber associated with amides and/or deoxyribose (1180–1190 cm^−1^) but retained variations in the levels of lipids (Table 1), further suggesting that the impairment of the YcaO protein can affect the cellular lipidome or interact with lipids. Lastly, of the four identified regions associated with variation for Δ*medM1M2M3*, two of those (1050, 1373–1380 cm^−1^) may be associated with the deletion of the corresponding genes (Table 1). Moreover, changes in amides I and II were also observed on this day (1650–1550 cm^−1^) (Table 1). Overall, across all days of analysis, one of the differences identified involved the vibration related to nucleic acids (C-O-C from nucleic acids, phosphodiester bonds, and deoxyribose bonds, and C-N from nucleotide bases), which might be related to the gene deletions in the mutants. However, differences in the vibration of bonds present in lipids were also constant, suggesting that MedM1, MedM2, and MedM3 enzymes and YcaO’s cellular role might be directly or indirectly related to these biomolecules. 

## 4. Conclusions

Herein, we describe the quantification of genes putatively involved in the biosynthesis of RiPPs by haloarchaea. Such analysis is usually performed using biomass from cultures in broth. However, most RiPPs act as antimicrobials, and *H. mediterranei* antihaloarchaea activity was observed exclusively in agar cultures, which motivated us to use biomass from agar cultures. The results show that the genes encoding the YcaO domain containing protein and lanthipeptide synthetases are transcribed, especially *med*M2. Investigation of the relationship between *ycaO* and antihaloarchaea activity showed no impact when the gene was deleted. Thus, so far, there is no experimental evidence supporting that RiPPs BGCs found in *H. mediterranei* are associated with the production of antimicrobials and, therefore, might have other biological roles. Thus, we performed FTIR analysis to identify possible biomolecules affected by the absence of LanM or YcaO proteins. FTIR is a valuable tool for screening purposes as it allows the analysis of colonies, the spectra generated distinguish molecules according to their structure, and it is an easy-to-perform, sensible, and cheap analytical technique. The major differences between strains were observed after 6 days of growth, but some of them might be related to the generation of the knockout mutants (absorption of the phosphodiester bond and amide bonds). Additionally, the absorption of C=O stretching regions from lipids (1750–1740 cm^−1^) also varied in both mutants, indicating that they have some differences. This may not be related to the cell membrane, where lipids are the major components since, in Archaea, an ether bond connects the lipid to the glycerol instead of an ester bond, as in Bacteria. Other known compounds that have C=O with strong absorption within 1750–1735 cm^−1^ are beta-lactones [33]. Thus, FTIR opened up new perspectives for further studies that should focus on the biological roles of these proteins for haloarchaea.

## Figures and Tables

**Table 1:** Summary of the wavenumbers and their possible assignments that caused the observed distribution in the PCA analysis (Figure 7b,c), for each day of analysis and according to [32].

Time	Strain	Wavenumber (cm^−1^)	Functional Group Assignments
Day 1	Δ*medM1M2M3*	1150	Phosphodiester bonds
		1280	Amides; Nucleic acids
		1450	Lipids, including fatty acids; Amide III
		1550	Amide II
		1650	Amide I
Day 6	Δ*ycaO*	1137–1145	Phosphate and/or oligosaccharides
		1180–1190	Amides and/or deoxyribose
		1650	Amide I
		1740–1750	C=O from lipids
Day 14	WR510	1150	Phosphodiester bonds
	Δ*ycaO*	1430–1450	Polysaccharides and/or lipids, including fatty acids; Amide III
		1740–1750	C=O from lipids; C=C from lipids including fatty acids
	Δ*medM1M2M3*	1050	Phosphate; Oligosaccharides and/or C-O-C from nucleic acids and phospholipids
		1373–1380	C-N from guanines and/or cytosines; C-O and/or C-H and/or N-H
		1534	Amide II
		1550	Amide II
		1650	Amide I
		1740–1750	C=O from lipids

## Data Availability

Not applicable.

## Supplementary Materials

The following supporting information can be downloaded at: https://www.mdpi.com/article/10.3390/biology12020236/s1, Figure S1: Schematic summary of the construction of recombinant plasmids; Figure S2: Schematic view of the transformation of *H. mediterranei* WR510 and the selection of knockout mutants; Figure S3: Schematic representation of the assay applied to evaluate *H. mediterranei* WR510 growth and antihaloarchaea activity; Figure S4: Schematic representation of the general procedure used to prepare *H. mediterranei* dot plates for RNA extraction and FTIR analysis; Figure S5: Alignment of the genetic regions containing *ycaO* genes (red ORF) in different haloarchaea; Table S1: Composition of haloarchaea culture media; Table S2: List of primers and respective amplification parameters used for amplification of flanking regions for the construction of knockout plasmids, pTA131 screening of knockout plasmids, and pop-in transformants.
