# Supplementary material for: Towards the Understanding of the Function of Lanthipeptide and TOMM-Related Genes in Haloferax mediterranei
Source: Biology (Basel). 2023 Feb 2;12(2):236. doi: 10.3390/biology12020236 (PMC9953058; doi:10.3390/biology12020236)
Supplement: Supplementary file 1 [file biology-12-00236-s001.zip › biology-2133201-SI.pdf]

Supplementary Materials

Towards the Understanding of the Function of Lanthipeptide and TOMM-Related Genes in Haloferax mediterranei

Thales Costa 1,† , Elena Cassin 1,†, Catarina Moreirinha 2,* , Sónia Mendo 1 and Tânia Sousa Caetano 1,*

1 CESAM and Department of Biology, University of Aveiro, 3810-193 Aveiro, Portugal
2 CESAM and Department of Chemistry, University of Aveiro, 3810-193 Aveiro, Portugal
* Correspondence: catarina.fm@ua.pt (C.M.); tcaetano@ua.pt (T.S.C.)
† These authors contributed equally to this work.

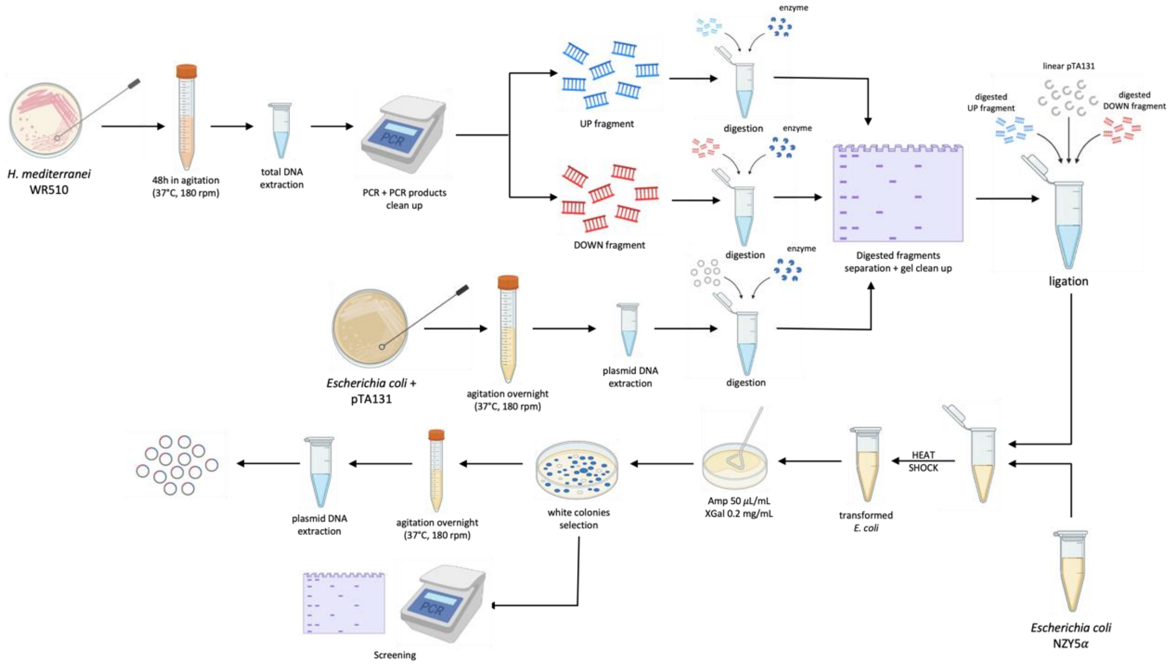

Figure S1: Schematic summary of the construction of recombinant plasmids.

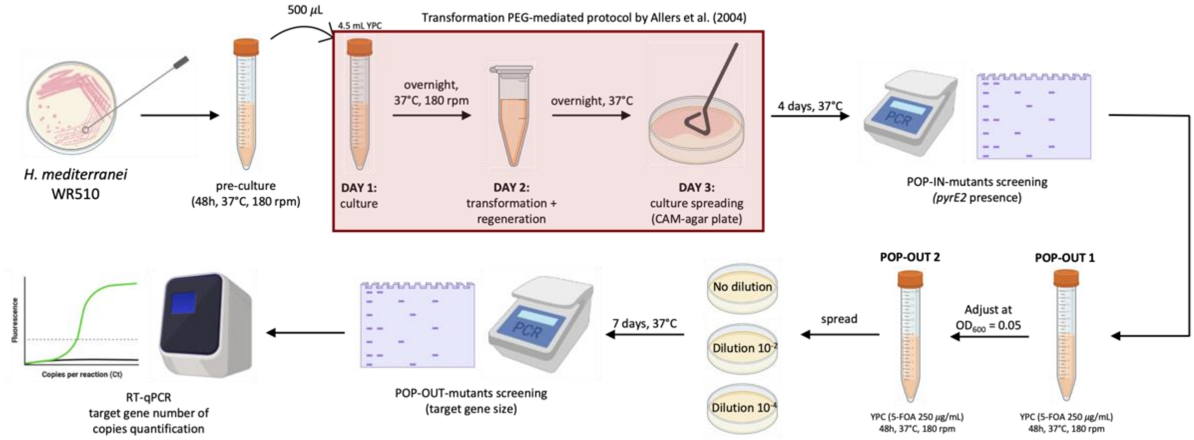

Figure S2: Schematic view of the transformation of *H. mediterranei* WR510 and the selection of knockout mutants.

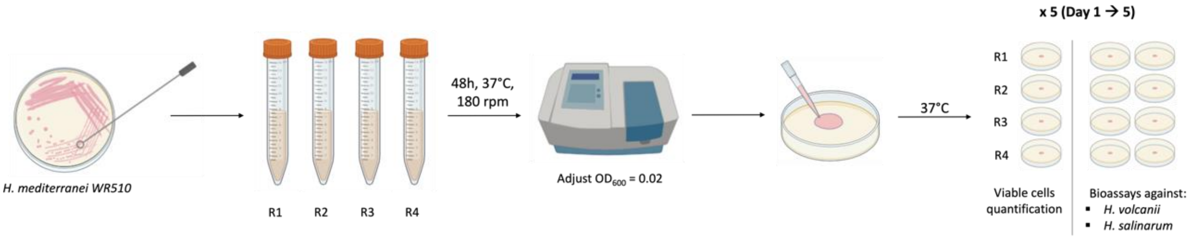

Figure S3: Schematic representation of the assay applied to evaluate *Haloferax mediterranei* WR510 growth and anti-haloarchaea activity.

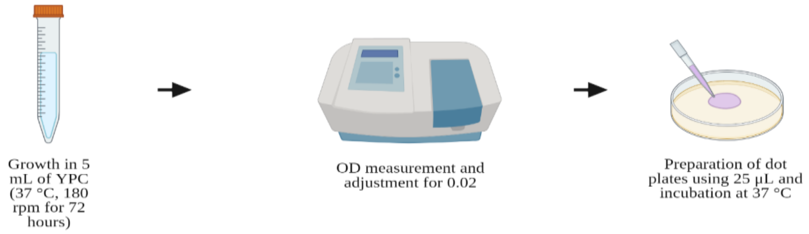

Figure S4: Schematic representation of the general procedure used to prepare *H. mediterranei* dot plates for RNA extraction and FTIR analysis.

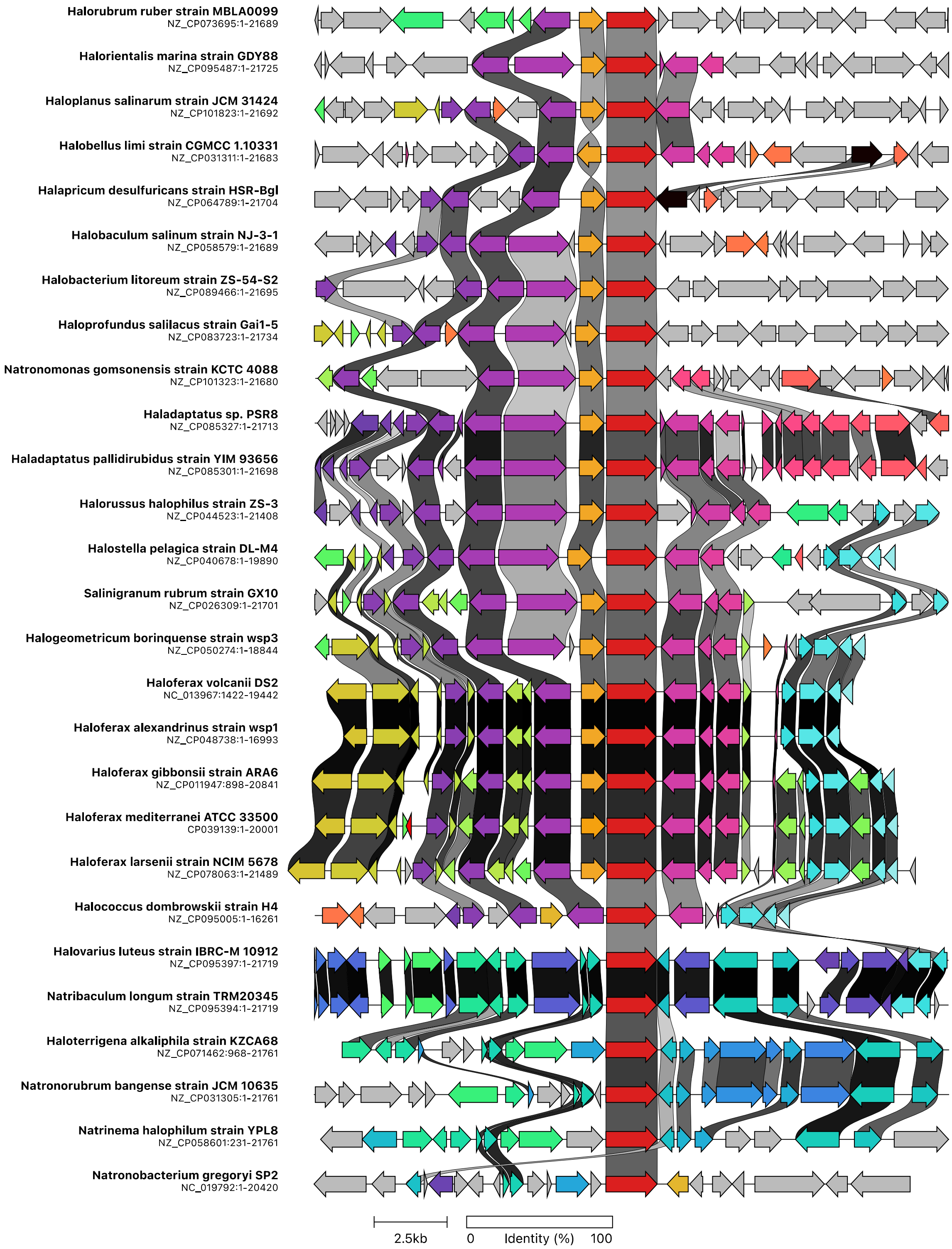

Figure S5: Alignment of the genetic regions containing *yca*O genes (red ORF) in different haloarchaea.

Table S1: Composition of haloarchaea culture media.

Solution or Medium	Composition (quantities per litre)	Notes
salt-water 30% (SW 30%)	240g NaCl<br>30g of $MgCl_2 \bullet 6H_2O$<br>35g $MgSO_4 \bullet 7H_2O$<br>7g of KCl<br>17.6 mL of a 1M TrisHCl solution (pH 7.5)	After preparation, the solution was auto-claved.
YPC 10X solution	50g of yeast extract (Liofilchem)<br>10 g peptone of meat (Merck)<br>10g of casamino acids (Difco Laboratories)<br>17.6 mL of a 1M KOH solution	After preparation, the solution was auto-claved.
CAM 10X solution	70g of casamino acids (Difco Laboratories)<br>17.6 mL of a 1M KOH solution	After preparation, the solution was auto-claved.
YPC (yeast-peptone-casamino acids) broth	98 mL of YPC 10X<br>598 mL of salt-water 30% (SW 30%)<br>298 mL of distilled water (dH_2O)<br>6mL of 0.5 M $CaCl_2$	All solutions were prepared and auto-claved separately, and then mixed together in a sterile environment. $CaCl_2$ solution was filter sterilized with a 0.22μm cellulose acetate filter (Frilabo).
YPCss (YPC super salted) broth	98 mL of YPC 10X<br>896 mL of salt-water 30% (SW 30%)<br>6mL of 0.5 M $CaCl_2$	All solutions were prepared and auto-claved separately, and then mixed together in a sterile environment. $CaCl_2$ solution was filter sterilized with a 0.22μm cellulose acetate filter (Frilabo).
CAM (casamino acids) broth	98 mL of CAM 10X<br>598 mL of salt-water 30% (SW 30%)<br>298 mL of distilled water (dH_2O)<br>6mL of 0.5 M $CaCl_2$	All solutions were prepared and auto-claved separately, and then mixed together in a sterile environment. $CaCl_2$ solution was filter sterilized with a 0.22μm cellulose acetate filter (Frilabo).
YPC-agar/YPCss-agar/CAM-agar<br>(1.5% agar)	YPC broth/YPCss broth/ CAM broth, 15g of bacteriological agar (Liofilchem)	The corresponding broth was warmed up and 15g of agar per litre were added.
YPC-soft-agar/YPCss-soft-agar<br>(1% agar)	YPC broth/YPCss broth, 10g of bacteriological agar (Liofilchem)	The corresponding broth was warmed up and 10g of agar per litre were added.

Table S2: List of primers and respective amplification parameters used for amplification of flanking regions for the construction of knockout plasmids, pTA131 screening of knockout plasmids and pop-in transformants.

Target	Primers		Restriction enzyme	Annealing Temperatue
	Designation	Sequence (5'à3')		
ycaO up region	ycaO_UP_FW	GAC**TCTA-GA**GGTACTCGGTTCGCTCTCAT	*Xba*I	69ºC
	ycaO_UP_RV	TAA**GCGGCCGC**GGTCCCCGCAATATCGCC	*Not*I	
ycaO down region	ycaO_DOWN_FW	ATT**GCGGCCGC**CAGACGCTGCTCAGACATTC	*Not*I	69ºC
	ycaO_DOWN_RV	CAT**GGATCC**GGCAACAACCTCATTCCGAA	*Bam*HI	
lacZ MCS	pUC19_FW	AGGGTTTTCCCAGTCACGAC	-	54ºC
	pUC19_RV	CTTCCGGCTCGTATGTTG	-	
ycaO (qPCR)	check_ycaO_qPCR_Fw	GAGTTGGACGACGCACTCTC	-	61 ºC
	check_ycaO_qPCR_Rv	TGCGACATAGTACGGAAGCG	-	
medM1 (qPCR)	qPCR_medM1_fw	ACGATTATCGATGCGGAGAC	-	59 ºC
	qPCR_medM1_rv	GGTCATCACGTCTGTGTTGG	-	
medM2 (qPCR)	qPCR_medM2_fw	GAAGCCGTTCGAAACTGAG	-	58 ºC
	qPCR_medM2_rv	ACGGTGTGTTGGTGTATGGA	-	
medM3 (qPCR)	qPCR_medM3_fw	TTGCATTGCGTATCTGCTTC	-	59 ºC
	qPCR_medM2_rv	GCTGGTCGTCTTCTCTGGAC	-	
rpl16 (qPCR)	qPCR_rpl16s1_F	CCACGTCATCCGCGAGAACA	-	57 ºC
	qPCR_rpl16s1_R	CGACCTTCCCGAACGACTGG	-	
